# One prospective study of physiologic sea saline nasal care on the incidence of acute upper respiratory tract infections and changes of pharyngeal pathogens in children during kindergarten daycare

**DOI:** 10.1186/s12887-025-06424-8

**Published:** 2025-12-12

**Authors:** Chunchou Luo, Yanzhen Yang, Qiong Fang, Shuyun Zhang, Xiumei Lin, Xiangui Chen, Ping Zhang

**Affiliations:** 1 Nursing Department, Zhangzhou Affiliated Hospital of Fujian medical University, Zhangzhou, Fujian China; 2 Pediatric Respiratory Medicine Department, Zhangzhou Affiliated Hospital of Fujian medical University, Zhangzhou, Fujian China; 3 Otolaryngology Department, Zhangzhou Affiliated Hospital of Fujian medical University, Zhangzhou, Fujian China; 4 Pediatric Outpatient Clinic, Zhangzhou Affiliated Hospital of Fujian medical University, Zhangzhou, Fujian China; 5 National Children’s Medical Center, Children’s Hospital of Fudan University, Shanghai, China

**Keywords:** Physiologic sea saline, Nasal spray irrigation, Preschool, Pharyngeal pathogen, Acute upper respiratory tract infection

## Abstract

**Objective:**

To explore the effects of physiologic sea saline nasal spray irrigation on the incidence of acute upper respiratory tract infections (AURTIs) and pharyngeal pathogens during children’s admission to child care, and to provide a theoretical basis for health care in kindergartens.

**Methods:**

Healthy children enrolled in a public kindergarten in the region from February to June 2023 were divided into the intervention group and the control group by using cluster random allocation concealment method. The intervention group was given physiological sea saline nasal care twice a day, while the control group received no special intervention. Swab samples were collected for pathogen detection at the time of admission (baseline) and at the time of the first occurrence of AURTIs more than 48 h after admission. The incidence of AURTIs throughout the entire semester of enrollment and the trend of dispersion in different months were compared between the two groups, and the differences of pharyngeal pathogens (bacterial species, colony counts and non-bacterial pathogens) after the first AURTIs during the kindergarten period were compared with the baseline.

**Results:**

A total of 234 children who met the inclusion and exclusion criteria were included in the analysis, with 120 children in the intervention group and 114 children in the control group. The incidence of AURTIs in the intervention group was significantly lower than that in the control group (45.83% vs. 71.05%, *x*^*2*^ = 15.276, *P* < 0.001), and the occurrence time of the first AURTIs was significantly more dispersed than that in the control group (*Z* = 25.075, *P* < 0.01). The number of bacterial species and colonies cultured in the throat swab of children in the intervention group were significantly less than those in the control group after the onset of AURTIs for the first time (*t* = 8.591, *P* < 0.001; *x*^*2*^ = 8.432, *P* = 0.015). Compared with the baseline, the number of pharyngeal bacteria species in the intervention group decreased after the first occurrence of AURTIs, while that in the control group increased, with statistical significance (*t* = 6.426, *P* < 0.001; *t* = 3.28, *P* = 0.001). There was no significant difference between the two groups in the detection rates of viruses and mycoplasma (*x*^*2*^ = 0.251, *P* = 0.616; *x*^*2*^ = 0.071, *P* = 0.790).

**Conclusions:**

Physiologic sea saline water nasal irrigation care can reduce the bacterial loads of pharynx colonization after the onset of AURTIs, reduce the incidence and concentrated incidence trend of AURTIs, but has no effect on the detection rate of non-bacterial pathogens such as viruses and mycoplasma.

## Introduction

Acute upper respiratory tract infections (AURTIs) are one of the most common diseases in infants and children. Pathogenic microorganisms such as bacteria, viruses and mycoplasma can cause the disease alone or mixed infection. Since pathogens can be transmitted through droplets or toys, environmental factors are related to the occurrence of AURTIs [1]. Changes in the environment, diet, and living habits caused by children living in groups after entering daycare can also lead to a concentrated incidence of AURTIs [2]. Physiologic sea saline nasal irrigation is widely used as an adjuvant treatment for colds, chronic sinusitis and allergic rhinitis, with good efficacy and relative safety in clinical use [3, 4]. Previous research on this topic has shown that admission to kindergarten group living can cause changes in the colonization of pathogens in the nasopharynx, leading to the occurrence of AURTIs [5]. Meanwhile, based on the safety of physiological sea saline nasal irrigation, this study used whole cluster randomized sampling to investigate the effects of daily nursing interventions of physiological sea saline nasal irrigation on pharyngeal colonization flora and etiology of AURTIs in healthy children, so as to provide a theoretical basis for health care in kindergartens.

## Methods

### Ethics

This study was approved by the Ethics Committee of Zhangzhou Municipal Hospital Affiliated to Fujian Medical University (Ethics No. 2022KYB121).And the informed consent forms for participation are received from the guardians.

## Participants

Healthy preschool children attending the Longwen District Government Kindergarten in Zhangzhou City, Fujian Province, who met the specified inclusion and exclusion criteria were enrolled in the study from February to June 2023 [6]. Inclusion criteria: ① No symptoms of AURTIs such as runny nose, sore throat, cough and fever in the past week; ② No use of antimicrobials in the past week; ③ No acute or chronic diseases including dyspepsia, primary immunodeficiency diseases and other related underlying diseases with previous diagnosis; ④ Guardians of participants signed informed consent forms. Exclusion criteria: ① Diagnosed as rhinitis, sinusitis, deviated nasal septum or leukemia epistaxis; ② Failure to complete the national immunization schedule in accordance with the time due to physical or other reasons.

### Sample size

The sample size was calculated according to the formula N = π_0_(1-π_0_)* [(u_α_+u_β_)/δ]^2^ [7]. It is stipulated that α = 0.05 and β = 0.1, unilateral u_0.1_=1.282 and unilateral u_0.05_=1.645. The incidence of AURTIs in healthy preschool children in the first semester after admission to public kindergartens in this region is about 75% [5], and it is estimated that physiologic sea saline water nasal irrigation care can reduce the incidence of AURTIs to 60%. According to the formula, the sample size of the intervention group was about 75 cases. Considering the convenience of drop-out and random sampling of the whole group, the sample size was increased to 100 cases per group.

### Interventions

Intervention group: Before the study began, strict training and hygiene guidance were provided to the relevant personnel, who were taught the correct operational methods and precautions according to the Expert Consensus on the application of nasal saline irrigation in Children with Upper Respiratory Tract Infections [8]. Nasal spray care with physiological sea saline was administered twice a day (once before entering kindergarten and once before leaving kindergarten) until the end of the semester. During the procedure, participants stood with their heads slightly tilted back, while the operator stood to the right front of the participant, placed the nozzle of the physiological sea saline in front of the nostrils, and gently pressed the manual pump to deliver the solution in a mist form. Four sprays were administered into each nostril at a time. The operator then gently pinched the nostrils bilaterally, wiped away nasal secretions with a tissue, and repeated the nasal cavity cleaning process as long as the children tolerated and cooperated. Care was taken to avoid excessive head tilting to prevent aspiration, and any associated discomfort was closely monitored and documented. Control group: The control group will not receive any special treatment.

### Randomization

This study adopted a cluster randomization method and used opaque sealed envelopes with class numbers, randomly assigned according to a random number table, and enrolled in a 1:1 ratio.

### Diagnostic criteria and management of aurtis

Acute upper respiratory tract infection refers to a general term for acute inflammation of the nasal cavity, pharynx, or throat [6]. The symptoms are as follows: (1) Common cold is dominated by nasopharyngeal catarrhal symptoms, which may include sneezing, nasal congestion and runny nose. (2) The main symptoms of acute pharyngitis are pharyngeal dryness, burning sensation and sore throat. (3) The symptoms of acute tonsillitis are mainly sore throat, fever and difficulty in swallowing. AURTIs can be accompanied by varying degrees of systemic symptoms, such as muscle pain, headache, chills, sweating and fatigue [9]. 

If the preschoolers in the two groups experienced the above symptoms of AURTIs 48 h after enrollment, the preschool institution would record the relevant symptoms of the preschoolers and report it to the research group, and notify them to go to the designated health care institutions for medical treatment, and carry out a second sampling.

### Pathogenic microorganism detection methods

Posterior pharyngeal wall specimens were collected by trained researchers. Before collection, the oral cavity was cleaned with physiological saline, tongue was pressed with tongue depressor, and the secretions of posterior pharyngeal wall were wiped twice with sterile swab and then the specimens were taken out and put into the specimen collection tube, and immediately stored in the refrigerator at 4℃. All specimens were collected on the same day and sent to the laboratory for testing [10]. 

The pharyngeal swab specimens were inoculated in blood agar plates and chocolate plates, and incubated in a 5% CO2 incubator at 35℃ for 18–24 h to observe the growth of pathogens, and bacteria were identified. Semi-quantitative analysis was performed using zonal demarcation methods, with 3+, 2+, and 1 + denoting large, medium, and small amounts, respectively [11]. Nucleic acid detection of 13 respiratory pathogens including influenza A virus, influenza B virus, parainfluenza virus, respiratory syncytial virus, adenovirus, rhinovirus, human metapneumovirus, Boca virus, coronavirus (229E, NL63, HKU1, OC43), COVID-19, Mycoplasma pneumoniae and Bordetella pertussis was performed by fluorescent label PCR(Hongshi SLAN-96 S Real-Time Fluorescent Quantitative PCR Instrument).PCR-fluorescent probe method was used for DNA quantitative detection of Chlamydia pneumoniae nucleic acid.

### Quality control

Specimens were sent for testing immediately after collection and transported at room temperature for less than 2 h to avoid bacterial death due to drying. Specimens with delayed delivery were placed in a 4℃ refrigerator and tested within 24 h [12]. All specimens were tested by the same pathogenicity testing biological company to ensure the quality of testing. The physiological sea saline used in the intervention group was assigned a corresponding number and placed in a fixed location. Two nurses from the research group and kindergarten healthcare doctors were required to perform daily operations and hand hygiene disinfection before and after operation to prevent cross-infection. The main researchers conducted irregular flight inspections, and problems were promptly recorded and rectified, and the study was stopped if necessary.

### Statistical methods

SPSS 22.0 software was used for statistical analysis. T-test was used for quantitative data, and the chi-square test or Fisher’s exact probability method, rank-sum test were used for count data, with a test level of a = 0.05. Median description was used for skewed distribution data.

## Results

The intervention group completed the study with 120 children and the control group completed the study with 114 children. There were no statistically significant differences between the two groups in gender, age, weight, actual school days per capita, or detection of pharyngeal pathogens at the time of enrollment (Table 1).

**Table 1:** Comparison of general information and first pharyngeal pathogen detection between two groups

	Intervention group	Control group	t value/Χ^2^ value	*P* value
Gender(male/female)	58/62	56/58	0.015	0.904
Age(year)	4.78 ± 0.32	4.77 ± 0.28	0.212	0.832
Weight(kg)	17.83 ± 0.92	17.95 ± 1.12	0.903	0.368
Actual school days	94.4 ± 1.89	94.06 ± 1.92	1.347	0.178
Bacterial count per capita(species)	2.86 ± 0.74	2.75 ± 0.76	1.063	0.289
Number of viruses detected (%)	6(5.0)	5(4.4)	0.040	0.824
Mycoplasma detections (%)	1(0.83)	2(1.75)	0.392	0.614
Chlamydia detections (%)	0	0	0	0

### Changes in the incidence of AURTIs and the discrete trend of the first onset time during the admission period of two groups

**Fig. 1:**
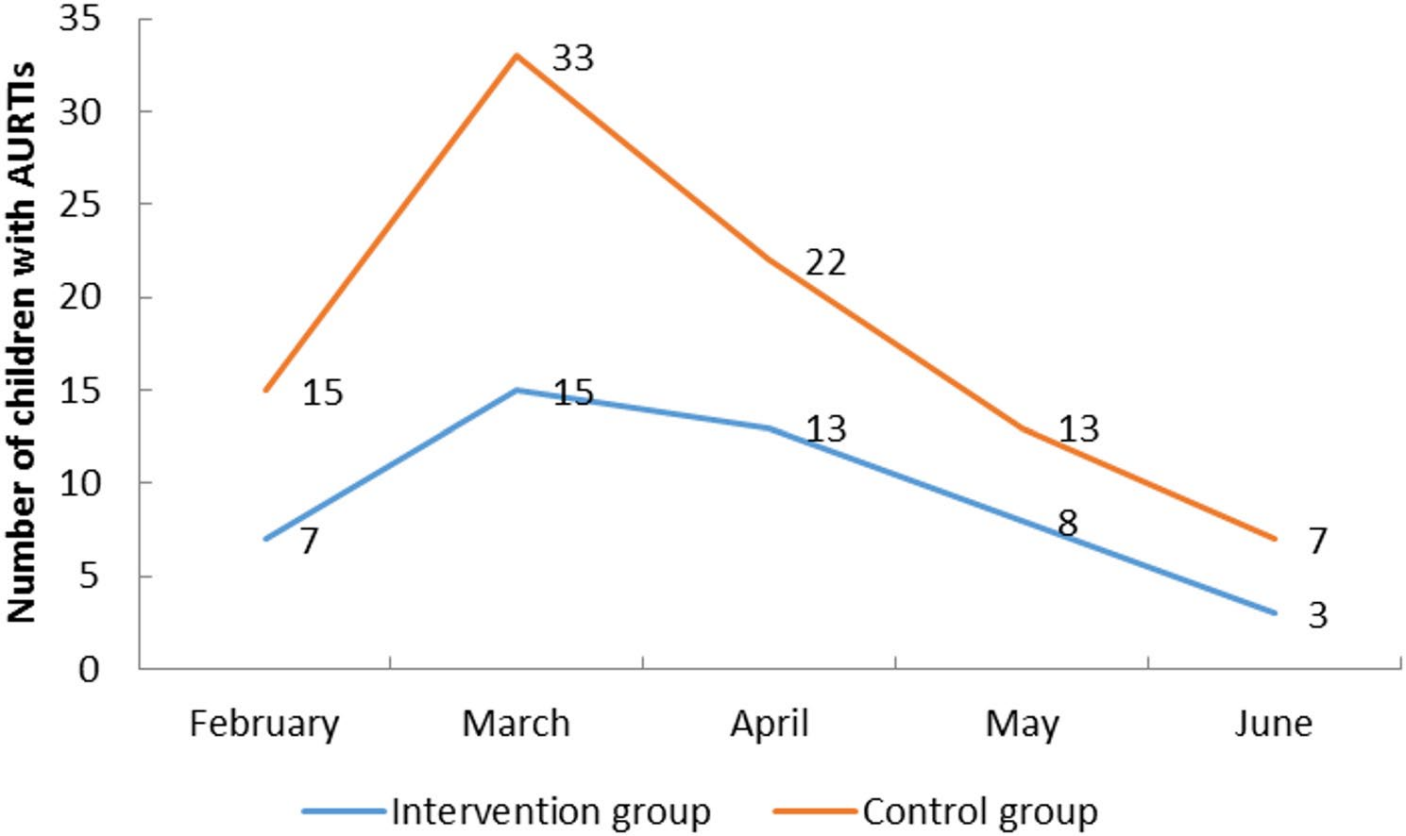
Trends in the number of children with first occurrence of AURTIs per month during childcare enrollment in both groups

The first occurrence of AURTIs in the control group was concentrated in February to April, while in the intervention group, the time of the first occurrence of AURTIs was relatively scattered (Fig. 1). There was a statistically significant difference in the number of children with upper respiratory tract infection for the first time in each month of the enrollment between two groups (*P* < 0.05).

### Changes in the number of bacterial species and colonies colonized in the pharynx of two groups before and after the first onset of AURTIs

There were statistically significant differences in the number of pharyngeal colonized bacteria species and colonies between the two groups in baseline and after the first onset of AURTIs (*P* < 0.05). The results of throat swab of one child in the control group showed there was no bacterial growth, which was not included in the statistics. The number of colonies was analyzed semi-quantitatively, with 3+, 2 + and 1 + representing large, medium and small amounts respectively, as shown in Table 2.

**Table 2:** Changes in the number of bacterial species and colonies colonized in the pharynx of children before and after the onset of the first AURTIs in both groups

		Intervention group	Control group	t value	*P* value
Baseline average bacterial species (species)^*^		2.86 ± 0.74	2.75 ± 0.76	1.063	0.289
Average number of pathogenic bacteria (species)^#^		2.15 ± 0.59	3.10 ± 0.66	8.591	<0.001
*t* value		*t* = 6.426	*t* = 3.287	-	-
*P* value		<0.001	0.001	-	-
Baseline bacterial colony count^*^	1+	135	131	0.187	0.911
	2+	156	141		
	3+	46	42		
Number of bacterial colonies after onset^#^	1+	61	99	8.432	0.015
	2+	49	111		
	3+	8	41		
*Χ*^*2*^ value*/Z* value		6.663	1.024	-	-
*P* value		0.036	0.599	-	-

* At baseline, there were 120 cases in the intervention group and 114 cases in the control group

^#^After the onset of AURTI, there were 55 cases in the intervention group and 81 cases in the control group

### Detection of non-bacterial pathogens after the first onset of AURTIs in two groups

The detection rates of virus and mycoplasma at the first onset of AURTIs in the two groups were significantly higher than the baseline. Two children in each group showed positive results for both viruses and mycoplasma. There was no significant difference in the detection rate of mycoplasma and virus in the first onset of AURTIs between the two groups (*P* > 0.05). In terms of the total detection rate of all other pathogens except bacteria, the difference was statistically significant (*P* < 0.05), as shown in Table 3.

**Table 3:** Comparisons of non-bacterial pathogens after the first onset of AURTIs between two groups

	Intervention group (*n* = 55)	Control group (*n* = 81)	Χ^2^ value	*P* value
Virus positive (%)	9(16.36)	16(19.75)	0.251	0.616
Mycoplasma positive (%)	14(25.45)	19(23.46)	0.071	0.790
Chlamydia positive (%)	0	0	0	0
Non-bacterial positive (%)^*^	21(38.18)	33(40.74)	0.090	0.765

Two children in each of the intervention and control groups were positive for both viruses and mycoplasma, and when combined, they were deducted

## Discussion

### Physiologic sea saline nasal spray irrigation care can effectively reduce the incidence of AURTIs in healthy preschoolers during the period of care, and can avoid the concentrated incidence

Kindergarten provides an environment for children to contact and interact with other children, so that they can gradually adapt to the collective life. However, children are more exposed to respiratory pathogens, leading to a significant increase in the incidence of AURTIs due to the changes of diet, exercise and sleep after admission to the kindergarten [5]. 

The pharynx is the site of respiratory colonization and pathogen retention. Due to its connection with the nasal cavity, children can transmit pathogenic pathogens to the public environment through coughing, sneezing, and other means, causing group diseases. Gao Z et al. [13] found that the cluster incidence of AURTIs in a kindergarten amounted to 80.39% (41/51), of which 18 kinds of respiratory pathogens were found to be associated with rhinovirus infection. Ou ZX et al. [14] found that Streptococcus pneumoniae can cause short-term cluster disease in kindergarten children under certain conditions. At present, there are few studies on nasal care for healthy preschool children to prevent AURTIs. Literature reports that gargling can effectively reduce the number of days of absence from school due to AURTIs in preschool children, and good personal hygiene helps to reduce the occurrence of AURTIs in children [15]. 

Physiologic sea salt water nasal spray irrigation is widely used in the adjuvant treatment of colds, chronic sinusitis and allergic rhinitis, with good efficacy and relative safety in clinical use. Whether it is helpful to reduce the incidence of AURTIs during kindergarten by removing nasal secretions and colonizing pathogens through physiological sea saline nasal spray irrigation remains to be studied.

In this study, a cluster random sampling control study was used to investigate whether physiologic sea saline nasal spray irrigation care can reduce the incidence of AURTIs and avoid the clustering of AURTIs in children. The results of the study showed that the incidence of AURTIs during the whole semester was 45.83% (55/120) in the intervention group and 71.05% (81/114) in the control group, and the difference between the two groups was statistically significant (***Χ***^***2***^ = 15.276, *P* ≤ 0.001). The first incidence of AURTIs in the control group during the period of day-care was concentrated in the months of February-April, with an incidence rate of 86.42% (70/April). The incidence rate was 86.42% (70/81), whereas the first incidence of AURTIs in the intervention group was relatively average between different months, and the difference between the two groups was statistically significant (*Z* = 25.075, *P* < 0.001).

### Physiologic sea saline nasal spray irrigation can effectively reduce the number of bacterial colonies and bacterial species in the pharynx of children

As the respiratory system is one of the organs that have the most contact with the external environment, the upper respiratory tract has become the site where bacterial colonization is most likely to occur, and the types of colonizing bacteria vary in different regions and at different times. Coughtrie AL et al. [16] found that common colonizing bacteria in the nasal cavity and nasopharynx of British preschoolers (0–4 years old) were Streptococcus pneumoniae, Haemophilus influenzae, Catamorium spp. Staphylococcus aureus, etc. Hou A et al. [17] found that the pharyngeal colonizing bacteria in healthy children in Beijing were mainly streptococcus pneumoniae, haemophilus influenzae, group A type B hemolytic streptococcus, Staphylococcus aureus, Staphylococcus epidermidis, etc. Our previous study showed that the first occurrence of AURTIs in daycare children was accompanied by an increase in the number of common respiratory colonization pathogens, suggesting nasopharyngeal colonization bacteria were involved in the occurrence of AURTIs [18]. 

This study showed that two groups of healthy preschool children with rhinitis were infected with more than 10 types of bacteria at the time of admission, including Neisseria, Streptococcus aeruginosa, Micrococcus, Streptococcus pneumoniae, and Haemophilus influenzae. The average bacteria species were detected (2.81 ± 0.65) species in the intervention group and 202 strains of 2 + or more bacterial colonies, while the control group of (2.75 ± 0.76) species and 183 bacterial colonies with 2 + or more, and there was no significant difference in bacterial species and colonies between the two groups. After the first onset of AURTIs, the average number of bacteria detected in the pharynx of 55 children in the intervention group was (2.15 ± 0.59) species, with 57 strains of 2 + or more bacterial colonies, while the average number of bacteria detected in the pharynx of 81 children in the control group was (3.10 ± 0.66) species, with 152 strains of 2 + or more bacterial colonies, which was statistically significant between the two groups. The reason for the difference between the two groups may be due to the fact that the children in the intervention group were treated with physiological sea saline spray irrigation twice a day, which mechanically effectively removes secretions and colonized bacteria in the nasal cavity, reduces the types of colonized bacteria and the number of colonies in the nasal cavity, and helps to maintain a normal nasal mucous membrane barrier and restores a normal micro-ecological environment in the nasal cavity, which can help to reduce the incidence of AURTIs.

### Physiologic sea saline nasal spray irrigation fails to reduce the number of nonbacterial pathogens in the pharynx after AURTIs in children

In 2023, there have been outbreaks of respiratory diseases such as Mycoplasma pneumoniae infection and influenza A virus infection in China, which are partly caused by the fact that influenza virus, rhinovirus, human metapneumonovirus, adenovirus and mycoplasma pneumoniae are all common respiratory infection pathogens in winter and spring, with cyclical epidemics [19]. Due to the public concern caused by the novel coronavirus infection and the strengthening of personal protective measures against respiratory tract infections, China has experienced a low level of respiratory tract infectious diseases for nearly three years, causing the antibody level of the population to “ebb” reaction, and the number of people susceptible to respiratory tract diseases to increase significantly, resulting in a high prevalence trend of various respiratory tract diseases this year [20]. 

In this study, 6 cases (5.0%) of virus and 1 case (0.83%) of mycoplasma were detected on pharyngeal swabs of 120 healthy children in the intervention group when they were admitted to kindergarten, while 5 cases (4.4%) and 2 cases (1.75%) were detected on pharyngeal swabs of 114 children in the control group, which had no significant difference between the two groups. At the first occurrence of AURTIs, throat swabs from 2 children in each group were positive for both virus and mycoplasma. Non-bacterial pathogens were detected 23 times in 21 of 55 (17.5%) children in the intervention group, including 14 cases of mycoplasma, 4 cases of rhinovirus, 2 cases of coronavirus, and 1 case each of influenza A virus, human metapneumovirus, and influenza B virus, while 35 non-bacterial pathogens were detected in 33 of 81 (40.74%) children in the control group, including 18 cases of mycoplasma, 7 cases of rhinovirus, 4 cases of coronavirus, and 3 cases of influenza A virus, and 2 cases of novel coronavirus. The detection of chlamydia was negative in both groups of children at the time of admission and the first onset. The twice daily physiological sea saline nasal spray irrigation care failed to reduce the positive rate of non-bacterial pathogens in the pharynx of children with AURTIs, which was considered to be related to the low carrying rate of non-bacterial pathogens in the nasal mucosa, and the acute onset of AURTIs is mostly due to contact with external pathogens, such as close contact with people with acute mycoplasma or viral infection causing AURTIs.

In summary, the physiologic sea salt water nasal spray irrigation care can reduce the pharyngeal colonizing bacterial loads after the onset of AURTIs during children’s daycare and decrease the incidence rate and concentrated incidence trend of AURTIs, but has no effect on the detection rate of non-bacterial pathogens such as virus and mycoplasma after AURTIs occurrence.

## Data Availability

No datasets were generated or analysed during the current study.
